# Gut microbiota-derived butyrate restores impaired regulatory T cells in patients with AChR myasthenia gravis via mTOR-mediated autophagy

**DOI:** 10.1186/s12964-024-01588-9

**Published:** 2024-04-03

**Authors:** Long He, Zhuotai Zhong, Shuting Wen, Peiwu Li, Qilong Jiang, Fengbin Liu

**Affiliations:** 1 Department of Digestive Endoscopy, The First Affiliated Hospital of Guangzhou University of Chinese Medicine, Guangzhou, China; 2 Guangdong Clinical Research Academy of Chinese Medicine, Postdoctoral Research Station of Guangzhou University of Chinese Medicine, No. 16 Airport Road, Baiyun District, Guangzhou, Guangdong Province 510405 China; 3 Department of Gastroenterology, Wangjing Hospital, China Academy of Chinese Medical Sciences, No. 6, Wangjing Zhonghuan South Road, Futong East Street, Chaoyang District, Beijing City, China; 4 Department of Gastroenterology, The Second Affiliated Hospital of Guangzhou University of Chinese Medicine, No. 55, Inner Ring West Road, Panyu District, Guangzhou, Guangzhou, Guangdong Province 511400 China; 5 Department of Hepatobiliary, The First Affiliated Hospital of Guangzhou University of Chinese Medicine, No. 16 Airport Road, Baiyun District, Guangzhou, Guangdong Province 510405 China; 6 Department of Myopathies, The First Affiliated Hospital of Guangzhou University of Chinese Medicine, No. 16 Airport Road, Baiyun District, Guangzhou, Guangdong Province 510405 China; 7 Baiyun Hospital of the First Affiliated Hospital of Guangzhou University of Chinese Medicine, No. 2 He Longqi Road, Renhe, Baiyun District, Guangzhou, 510000 China; 8 Institute of Gastroenterology, The First Affiliated Hospital of Guangzhou University of Chinese Medicine, No. 12 Airport Road, Baiyun District, Guangzhou, Guangdong Province 510405 China

**Keywords:** Myasthenia gravis, Treg, Gut microbiota, Butyrate, Autophagy

## Abstract

More than 80% of patients with myasthenia gravis (MG) are positive for anti-acetylcholine receptor (AChR) antibodies. Regulatory T cells (Tregs) suppress overproduction of these antibodies, and patients with AChR antibody-positive MG (AChR MG) exhibit impaired Treg function and reduced Treg numbers. The gut microbiota and their metabolites play a crucial role in maintaining Treg differentiation and function. However, whether impaired Tregs correlate with gut microbiota activity in patients with AChR MG remains unknown. Here, we demonstrate that butyric acid-producing gut bacteria and serum butyric acid level are reduced in patients with AChR MG. Butyrate supplementation effectively enhanced Treg differentiation and their suppressive function of AChR MG. Mechanistically, butyrate activates autophagy of Treg cells by inhibiting the mammalian target of rapamycin. Activation of autophagy increased oxidative phosphorylation and surface expression of cytotoxic T-lymphocyte-associated protein 4 on Treg cells, thereby promoting Treg differentiation and their suppressive function in AChR MG. This observed effect of butyrate was blocked using chloroquine, an autophagy inhibitor, suggesting the vital role of butyrate-activated autophagy in Tregs of patients with AChR MG. We propose that gut bacteria derived butyrate has potential therapeutic efficacy against AChR MG by restoring impaired Tregs.

**Supplementary Information:**

The online version contains supplementary material available at 10.1186/s12964-024-01588-9.

## Background

Myasthenia gravis (MG) is an autoimmune disorder characterized by weakness in the ocular, respiratory, limb, and bulbar muscles [1] that is worsened by activity [2]. Long-term studies have confirmed that muscle weakness in MG is typically caused by autoantibodies against nicotinic acetylcholine receptors (AChRs), muscle-specific tyrosine kinase, lipoprotein receptor-related protein 4, and agrin [3–6]. Anti-AChR antibodies are detectable in 80-85% of patients with MG [7, 8], while other subtypes of antibodies can be detected in only a small percentage of patients with MG. Thus, the presence of anti-AChR antibodies have become one of the significant clinical hallmarks of MG [9].

AChR-specific CD4^+^ T cells (T helper (Th) 1 and Th17 cells) and related cytokines [10, 11] are involved in the synthesis of anti-AChR antibodies [12]. Increased numbers of Th1 and Th17 cells stimulate B cells to produce excessive anti-AChR antibodies in patients with AChR MG [13–15]. However, the interaction of AChR-specific CD4^+^ T cells with B cells to produce anti-AChR antibodies is regulated by regulatory T (Treg) cells. Treg cells maintain immune homeostasis by suppressing excessive immune responses [16]. In AChR MG, Tregs can function as suppressors to inhibit the abnormal over-differentiation of AChR-specific CD4^+^ T cells and related cytokines [17, 18], thereby balancing the production of anti-AChR antibodies. Researchers have observed the impaired suppressive function of Tregs in AChR MG, and the proportion of Tregs is lower in patients with AChR MG than in healthy controls [19]. However, the mechanism of Treg dysfunction and reduced Treg cell numbers in AChR MG is not fully understood.

The gut microbiota play a significant role in the progression of various autoimmune diseases, such as rheumatoid arthritis [20], systemic lupus erythematosus [21], and multiple sclerosis [22]. Previous researches have demonstrated that the composition and abundance of gut microbiota in MG are significantly different from healthy controls [23]. Gut microbiota may serve as biomarkers for the diagnosis of MG [23]. Recent studies have confirmed that gut microbiota could potentially affect the host immune responses is by directly functioning as antigens or via their metabolites. For example, *Clostridia* and *Roseburia* are known to promote the numbers of Treg cells as well as contributing to the production of short-chain fatty acids (SCFAs) [24]. SCFAs, including acetic acid, propionic acid, and butyric acid, can activate the differentiation of CD4^+^ T cells [25, 26] and CD8^+^ T cells [27]. Moreover, reduced SCFAs levels, especially butyrate, have been observed in patients with MG [28]. However, disturbed gut microbiota as a characteristic of patients with AChR MG has not been reported, and the association between gut microbiota and impaired Tregs in AChR MG is still unknown.

In this manuscript, we analyzed the characteristics of gut microbiota in patients with AChR MG, and the serum levels of gut microbiota-derived SCFAs. We further investigated the effects of SCFAs, particularly butyrate, on Tregs in patients with AChR MG. This may provide a potential pathogenic mechanism for MG.

## Materials and methods

### Human subjects

11 healthy controls and 22 patients MG matched in gender and age were recruited. Patients were diagnosed according to the Myasthenia Gravis Foundation of America (MGFA). Patients were tested for antibodies using an enzyme-linked immunosorbent assay, and only patients with AChR antibody positive were included. All patients in our study were come from Guangdong province (China), and maintained in the same diet, and had not assume probiotics or antibiotics within three months (Table 1).

**Table 1:** Detailed clinical characteristics of all participants

Variables	HC	MG	*p*
Sample Sizes	11	22	-
Female	4(36.36%)	7(31.82%)	0.546
Age-years	49.55 ± 3.904	44.45 ± 2.770	0.2964
Duration of disease-years	-	3.50 ± 0.63	-
With thymoma	-	-	-
Anti-AChR antibody (+)	-	22(100%)	-
Immunosuppressive treatment	-	-	-
Inflammatory disease history (with the last months)	-	-	-
Antibiotic treatment (within the last 3 months)	-	-	-
MGFA classification			
IIa	-	2	-
IIb	-	20	-

Two-tailed student test for continuous variables (age), Chi-square analyses for categorical variables (sex)

### Sample collection

Fecal and peripheral blood samples were collected from HCs and patients with stage IIa to IIb AChR MG. Serum was obtained from fresh blood via centrifugation at 1500 g for 10 min, and peripheral blood mononuclear cells (PBMCs) were separated using human lymphocyte isolation solution (TBD Biological Manufacture Co., Ltd., Tianjin, China).

### Fecal genomic DNA extraction

Fecal samples were collected from HCs and patients with AChR MG. DNA extraction was performed on the samples using the MoBio DNA Isolation Kit (MO BIO Laboratories, Carlsbad, CA, USA).

### 16 S rRNA sequencing and bioinformatic analysis

Qualified genomic DNA samples underwent PCR amplification with the following primers: 515F (5’-GTGCCAGCMGCCGCGGTAA-3’ and 806R (5’-GGACTACHVGGGTWTCTAAT-3’). Amplification products were purified by Agencourt AMPure XP magnetic beads (Beckman Coulter Inc., Brea CA, USA). Agilent 2100 Bioanalyzer (Agilent Technologies, USA) was used to detect the resultant RNA fragment range and determine the concentration of the library. Qualified libraries were selected for sequencing on the HiSeq platform, based on the size of the inserted fragments. The raw sequencing data were processed using a window-based method to remove low-quality data. The paired reads obtained by paired-end sequencing were assembled into a sequence based on the overlapping relationship, and then FLASH software (Fast Length Adjustment of Short reads v1.2.11) was used to obtain Tags of the hypervariable region. The spelling was performed under the condition of 97% similarity, using the USEARCH software (v7.0.1090). The connected Tags were clustered, and finally the sequence of OUT was obtained. By comparing with the chimera database in the 16 S chimera database (v20110519), the chimeras generated by PCR amplification were removed using UCHIME software (v4.2.40). All tags were aligned into OUT sequences one by one using the usearch_global, and finally obtained the abundance value of OUT for each sample.

### SCFAs measurements

Serum SCFAs were measured using the gas chromatography coupled with mass spectrometry (Agilent GC 7890B/MSD 5977 A; Wilmington, USA). The standards of SCFAs were mixtures of acetic acid, propionic acid, butyric acid, valeric acid, and caproic acid. All standards were purchased from Sigma-Aldrich.

### Purification of human naive CD4^+^ T cells and differentiation of Tregs

Human naive CD4^+^ T cells were sorted from fresh PBMCs using an EasySep™ Human Naive CD4^+^ T Cell Isolation Kit (Stem Cell Technologies, UK) following the manufacturer’s instructions. For Treg differentiation, naive CD4^+^ T cells (2 × 10^5^ cells) were cultured for 3 days in the presence of plate-bound anti-CD3 (Tonbo Biosciences, USA), 5 μg/ml anti-CD28 (Tonbo Biosciences, USA), 3 ng/ml TGF-β1 (PeproTech, NJ, USA), and 100 IU/ml IL-2 (PeproTech, USA), with or without butyrate (0.2 mM), as previously described [29].

### Treg purification and expansion

Human Tregs were isolated from fresh PBMCs using an EasySep™ Human CD4^+^CD127_low_CD25^+^ Regulatory T Cell Isolation Kit (Stem Cell Technologies, Canada), following the manufacturer’s instructions. For Treg expansion, sorted Tregs were cultured in the presence of plate-bound anti-CD3, anti-CD28 (5 μg/ml), and IL-2 (300 IU/ml) antibodies for 4 weeks. The culture media were changed every 5–7 days. Harvested Tregs were used for western blot analysis.

### Treg suppression assay

CD4^+^CD25^−^ Tresps were labeled with 5 μM CFSE (Thermo Fisher Scientific, USA) and co-cultured with sorted Tregs (2 × 10^5^ cells) for 5 days with or without butyrate (0.2 mM) or chloroquine (20 μM). The proliferation of Tresps was calculated using the mean fluorescence intensity of CFSE^+^ cells, which was measured by flow cytometry.

### Flow cytometry

For surface markers, cells were stained with FITC-conjugated anti-CD4 (Tondo Biosciences, USA), APC-conjugated anti-CD25 (Tondo Biosciences, USA), and APC-conjugated anti-CTLA-4 (BioLegend, USA) antibodies in phosphate-buffered saline containing 1% fetal bovine serum. For intracellular maker, cells were fixed, permeabilized, and then stained with PE-conjugated anti-Foxp3 (eBioscience, USA). FACS experiments were performed using BD Accuri C6 or LSRFortessa (BD Biosciences, USA). Data were analyzed using FlowJo V10 software.

### Western blot

Expanded CD4^+^CD127_low_CD25^+^ Tregs were cultured with or without butyrate (0.2 mM). On day 6, cells were harvested and the western blot analysis was performed using the following antibodies (all purchased from Cell Signaling Technologies, USA): phospho-mTOR (Ser2448, #5536), phospho-p70S6K (Thr398, #9209), SQSTM1/p62 (#23,214), LC3B (#3868), and β-actin (#3700).

### Immunofluorescence

Immunofluorescence assays were performed as previously described [30]. Briefly, naive CD4^+^ T cells or Tregs were sorted on polylysine-coated slides. The cells were fixed, permeabilized, blocked, and incubated with diluted primary antibodies overnight at 4 °C. On the second day, cells were washed and stained with Alexa Fluor™ 568 (# A-11,077, Thermo Fisher Scientific, USA). Finally, the cells were treated with Invitrogen SlowFade Gold anti-fade reagent (Thermo Fisher Scientific, USA).

### Measurement of glycoPER and OCR

Sorted naïve CD4^+^ T cells were cultured under Treg polarizing conditions as described above and treated with or without butyrate (0.2 mM) for 3 days, harvested, and seeded in 24-well plates coated with 22.4 μg/mL Corning™ Cell-Tak Cell and Tissue Adhesive (Biocoat, USA) using a density of 3 × 10^5^ cells per well. OCR and glycoPER were measured using the Seahorse XF Mito Stress Test Kit (Agilent Technologies, USA) and Seahorse XF Glycolytic Rate Test Kit (Agilent Technologies, USA), respectively, following the manufacturer’s instructions.

### Induction of experimental autoimmune MG (EAMG) model in mice

The EAMG mice model was established as previously described [23, 31]. Briefly, female C57B/L6 mice, aged 8–10 weeks, were immunized by subcutaneous injection with 50 μg of R97-116 peptides (purity ≥ 95%, GL Biochem, Shanghai, China) in complete Freund’s adjuvant (Sigma-Aldrich), boosted on the 4th and 6th weeks with R97-116 protein in incomplete Freund’s adjuvant (IFA). Clinical scores for MG were graded as follows: grade 0, no muscle weakness, even after 30 consecutive paw grips; grade 1, normal at rest but weakness after 30 consecutive paw grips; and grade 2, weakness even at rest [32]. All experimental protocols were approved by the Animal Ethics Committee of Guangzhou University of Chinese Medicine (TCMF1-2021012).

### Butyrate treatment

Mice were treated with 200 mM sodium butyrate in drinking water as previously described [26]. Sorted naïve CD4^+^T cells or Treg cells were treated with sodium butyrate at concentrations of 0 μM, 100 μM, 200 μM and 400 μM in cell culture medium. The 200 μM butyrate treatment performed more efficiently on promoting naïve CD4^+^T cell differentiation into CD4^+^CD25^+^FOXP3^+^Tregs. Accordingly, 200 μM butyrate was used for all subsequent experiments.

### Open field experiment

Open field experiments were performed as previously described [23]. Mice were kept in a quiet room for 0.5 h before experimental measurements. Then, a single mouse was gently placed in the corner of an opaque box (40 × 40 × 40 cm^3^). Motor activities of the mice were recorded for 5 min using a camera, and motion tracks were visualized by Imagine J software (NIH, MD, USA).

### Enzyme-linked immunoassay (ELISA)

ELISA kits for IFN-γ, IL-17 A and TGF-β were purchased from Dogesce (Beijing, China). A mouse anti-R97-116 antibody ELISA kit was purchased from Jingmei Biological Engineering Co. Ltd (Shenzhen, China). Cytokine levels from serum or culture supernatant samples were measured according to the manufacturer’s instructions.

### Statistics

All data were presented as the mean ± standard deviation (SD) or median (M) with interquartile boundary values *(P25–P75)* and were analyzed using GraphPad Prism V9.3. Differences between the two groups were analyzed using unpaired Student’s t-tests or Mann-Whitney *U*-tests. One-way ANOVA was used to compare multiple groups. Statistical significance was set at *p* < 0.05.

## Results

### Butyric acid-producing bacteria and butyric acid concentration were reduced in patients with AChR MG

**Fig. 1:**
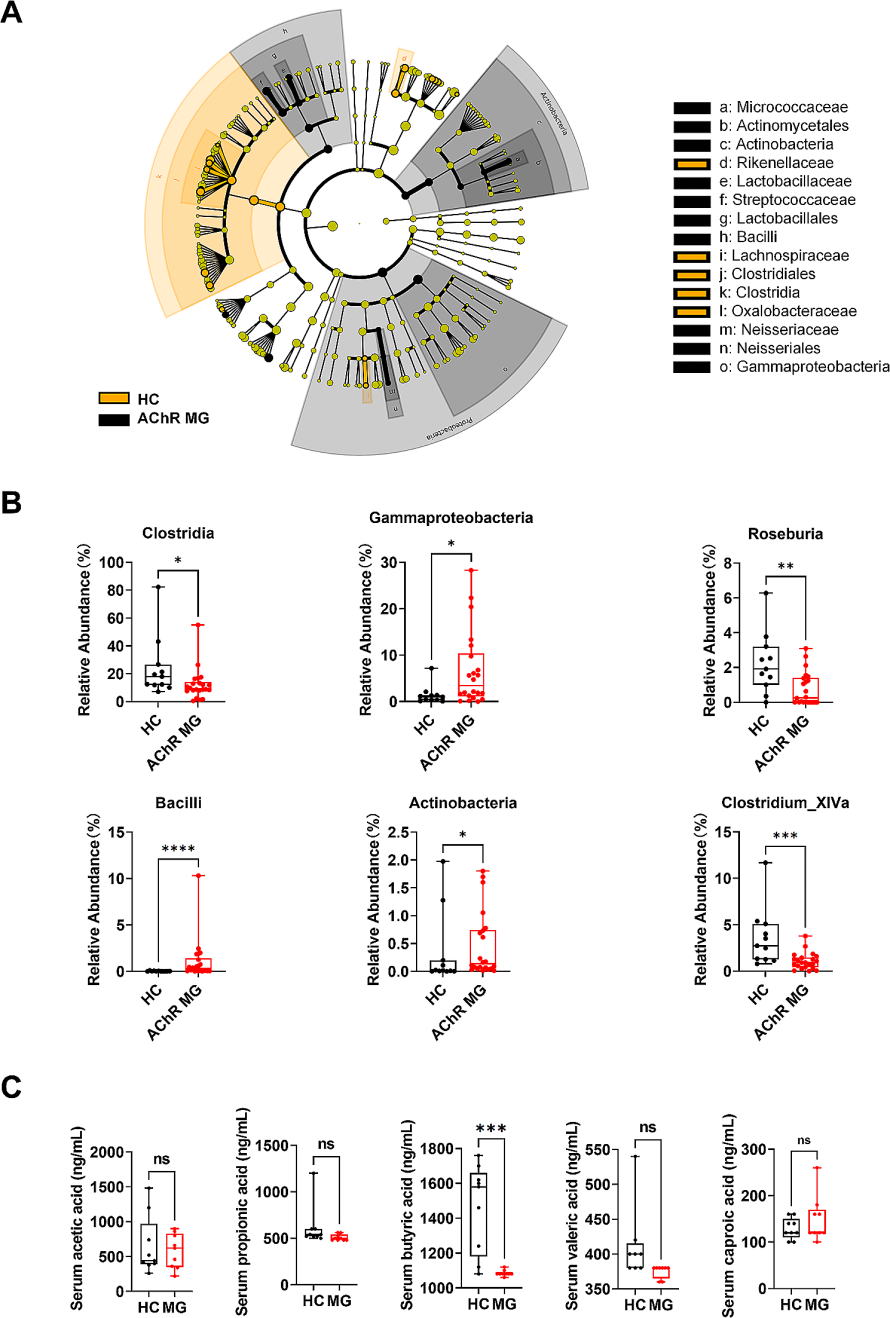
Profiling of the gut microbiota and short-chain fatty acids (SCFAs) in patients with AChR MG. (**A**) LEfSe clustering tree. Yellow color represents healthy controls (HCs, *n* = 11), and black color represents patients with AChR MG (*n* = 22). Nodes of different colors represent microbial groups that play an important role in the group represented by the color. (**B**) The relative abundance of *Clostridia* (Mann-Whitney U = 58, *p* = 0.0153), *Gammaproteobacteria (*Mann-Whitney U = 61, *p* = 0.0213), *Bacilli (*Mann-Whitney U = 25, *p* < 0.0001), *Actinobacteria* (Mann-Whitney U = 69, *p* = 0.0481), *Clostridium cluster _XlVa* (Mann-Whitney U = 37, *p* = 0.0008) and *Roseburia* (Mann-Whitney U = 54, *p* = 0.0094) in HCs compared to patients with AChR MG. (**C**) The concentrations of acetic acid, propionic acid, butyric acid (Mann-Whitney U = 5.5, *p* = 0.0008), valeric acid, and caproic acid in the sera of HCs and patients with AChR MG. Each dot represents an individual sample. * *p* < 0.05, ** *p* < 0.01, *** *p* < 0.001, ns = not significant

We recruited 11 healthy controls (HCs) and 22 patients with AChR MG. The detailed clinical characteristics of all participants are summarized in Table 1. Fecal samples were collected from these participants, and analyzed by the 16 S ribosomal RNA (rRNA) gene sequencing. Linear discriminant analysis of effect size identified significant differences in biomarker presence between the patients with AChR MG and HCSs. Compared with the AChR MG, *Clostridia*, *Clostridiales*, *Lachnospiraceae*, *Clostridium cluster _XlVa* and *Roseburia* were enriched in HCSs (Fig. 1A). Further, a disease classifier was constructed and 11 species were identified as candidate markers for AChR MG patients from HCs (Fig. S1A). The area under the receiver operating curve (AUC) of the corresponding ROC curve reached 0.959 (Fig. S1B). Moreover, the ROC curve of a validation cohort based on 12 HCs and 15 patients with AChR MG reached 0.811, which further highlighted the reliable role of these gut microbiota as a disease classifier between HCs and AChR MG (Fig. S1C). We also observed significant differences between HCSs and AChR MG at the class and genus levels. At the class level, the relative abundance of *Clostridia* was depleted, whereas that of *Gammaproteobacteria*, *Bacilli* and *Actinobacteria* was increased in patients with AChR MG (Fig. 1C). At the genus level, the relative abundances of *Clostridium* cluster *_XlVa* and *Roseburia* were lower in AChR MG (Fig. 1D).

Previous studies have reported that *Clostridia, Clostridium* cluster *_XlVa*, and *Roseburia* contribute to the production of SCFAs [24]. We quantified the concentrations of SCFAs in the serum of all participants and found that the butyric acid content in patients with AChR MG was significantly lower than that in HCs. No significant differences were observed in the concentrations of acetic acid, propionic acid, valeric acid, or caproic acid between the two groups (Fig. 1D).

### Butyrate enhances Treg differentiation and suppressive function

**Fig. 2:**
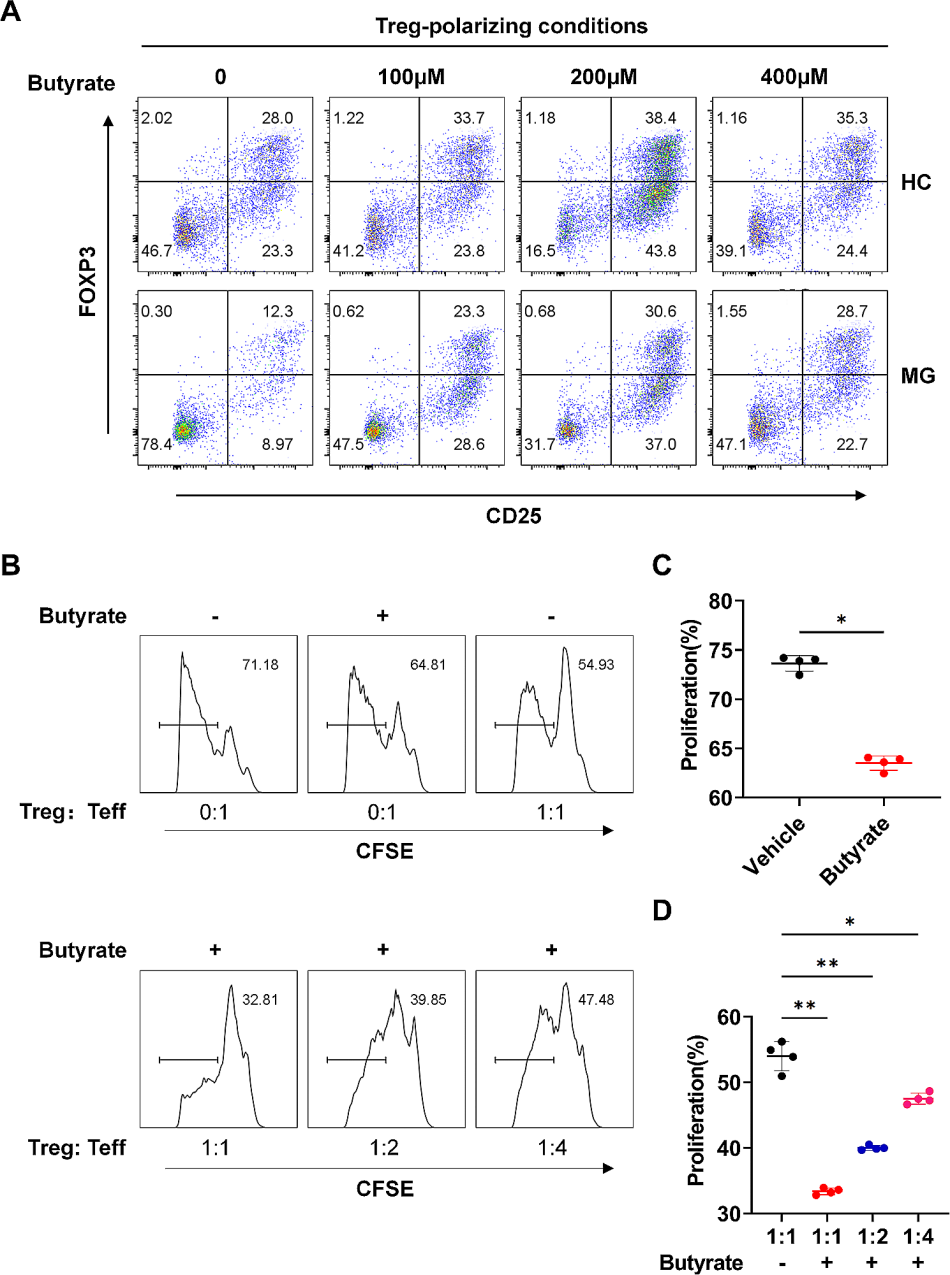
Butyrate promotes Treg differentiation and suppressive function. (**A**) Magnetically sorted naive CD4^+^ T cells (2 × 10^5^ cells) obtained from the peripheral blood of patients with AChR MG were cultured for 3 days under Treg-polarizing conditions using butyrate at concentrations of 0 μM, 100 μM, 200 μM and 400 μM. The frequencies of CD4^+^CD25^+^FOXP3^+^ Tregs were determined by flow cytometry. **(B, C, D)** Magnetically sorted CD4^+^CD25^−^ responder T cells (2 × 10^5^ cells) from patients with AChR MG were labeled with CFSE and cultured with CD4^+^CD25^+^CD127_low_ Tregs for 5 days in the presence of plate-bound anti-CD3 and anti-CD28, with or without 200 μM butyrate. The suppressive function of Tregs was measured by calculating the proliferation of CFSE^+^ cells (t = 18.81, *p* < 0.001for difference between Vehicle and Butyrate with unpaired *t*-test; F = 211.4, *p* = 0.0043 for difference between 1:1 and 1:2 with ANOVA, *p* = 0.0236 for difference between 1:1 and 1:4 with ANOVA). Each dot represents an individual sample. Data are shown as the mean ± SEM, * *p* < 0.05, ** *p* < 0.01, *** *p* < 0.001, ns = not significant

Next, we investigated the effects of butyrate on Treg differentiation and its suppressive function in AChR MG. Naïve CD4^+^T cells (sorted from HCs and patients with AChR MG) were cultured under Treg-polarizing conditions with different doses of sodium butyrate for 3 days. We observed that butyrate treatment performed excellently for promoting naïve CD4^+^T cell differentiation into CD4^+^CD25^+^FOXP3^+^Tregs (Fig. 2A) and significantly increased forkhead box P3 (FOXP3) protein expression in the naïve CD4^+^T cells (Fig. S2A). Accordingly, 200 μM of sodium butyrate was used for all subsequent experiments. In addition, Pearson’s correlation analysis showed that the serum butyrate concentrations in HCs and patients with AChR MG were positively correlated with Treg numbers, respectively (Fig. S2B).

To determine whether butyrate influences the Treg suppression function in patients with AChR MG, we co-cultured sorted Tregs with carboxyfluorescein succinimidyl ester (CFSE)-labeled CD4^+^CD25^−^ responder T cells (Tresps) in the presence of butyrate. After 5 days of culture, we observed that butyrate treatment directly inhibited the proliferation of CFSE-labelled Tresps (Fig. 2B, 2 C). Furthermore, Treg suppressive activity was significantly increased in the presence of butyrate at different ratios of Tregs versus Tresps (Fig. 2B and D).

### Butyrate reprograms the energy metabolism in Tregs

**Fig. 3:**
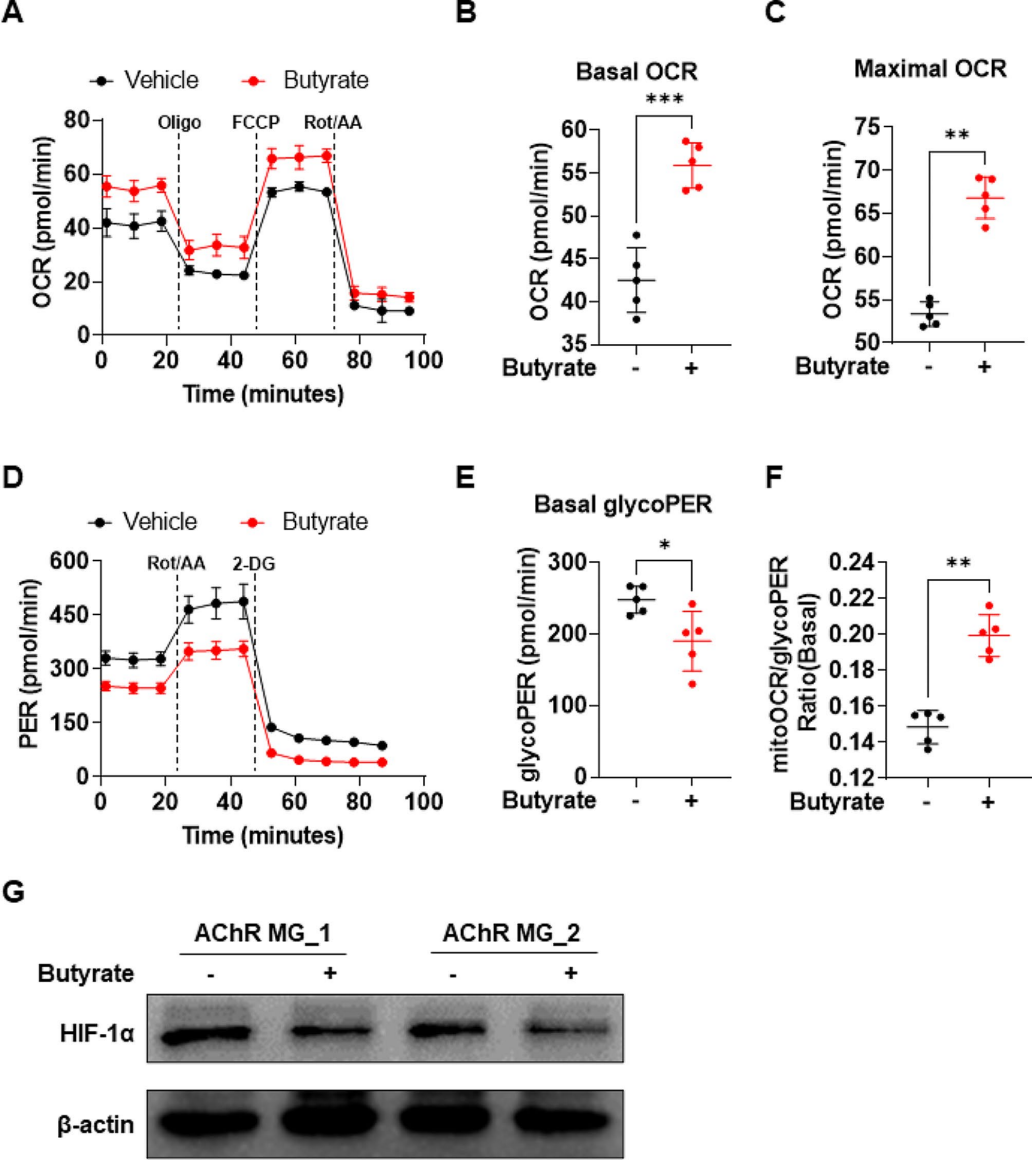
Butyrate treatment regulates the energy metabolism of Tregs in patients with AChR MG. Naive CD4^+^ T cells sorted from patients with AChR MG were cultured under Treg-polarizing conditions with or without 200 μM butyrate. (**A**) Mitochondrial oxygen consumption rate (mitoOCR) and (**D**) glycolytic proton efflux rate (glycoPER) were measured using a Seahorse XFe24 analyzer after 3 days of cell culture. Quantification of (**B**) basal OCR (t = 6.848, *p* = 0.0002 with unpaired *t*-test), (**C**) maximal OCR (t = 10.74, *p* = 0.0081 with unpaired *t*-test), (**E**) basal glycolysis (t = 2.826, *p* = 0.0223 with unpaired *t*-test), and (**F**) basal ratio of mitoOCR to glycoPER (t = 7.678, *p* = 0.0079 with unpaired *t*-test). (**G**) Protein expression of HIF-1α was detected by Western blot. Each dot represents an individual sample. Data are shown as the mean ± SD, * *p* < 0.05, ** *p* < 0.01, *** *p* < 0.001, ns = not significant

Oxidative phosphorylation (OXPHOS) and glycolysis metabolic pathways have been reported to regulate T cell differentiation. Thus, we assessed OXPHOS levels by measuring mitochondrial oxygen consumption rates (mitoOCR). Sorted naive CD4 + T cells obtained from patients with AChR MG were cultured under Treg-polarizing conditions in the presence of butyrate for 3 days. We observed that butyrate treatment significantly increased basal and maximum mitoOCR (Fig. 3A C). In addition, the glycolytic proton efflux rate (glycoPER) is the subtraction of the mitochondrial proton efflux rate from the total proton efflux rate [33] and is more representative of the glycolysis level than the extracellular acidification rate. We observed that butyrate supplementation significantly decreased glycoPER levels (Fig. 3D and E) and increased the basal mitoOCR/glycoPER ratio (Fig. 3F). An increased mitoOCR/glycoPER ratio indicates the preferential use of OXPHOS over glycolysis. Hypoxia-inducible factor-1 alpha signaling is known to promote glycolysis [34], we observed reduced expression of this protein under butyrate treatment (Fig. 3G).

### Butyrate enhances surface cytotoxic T-lymphocyte-associated protein 4 (CTLA-4) expression of Tregs

**Fig. 4:**
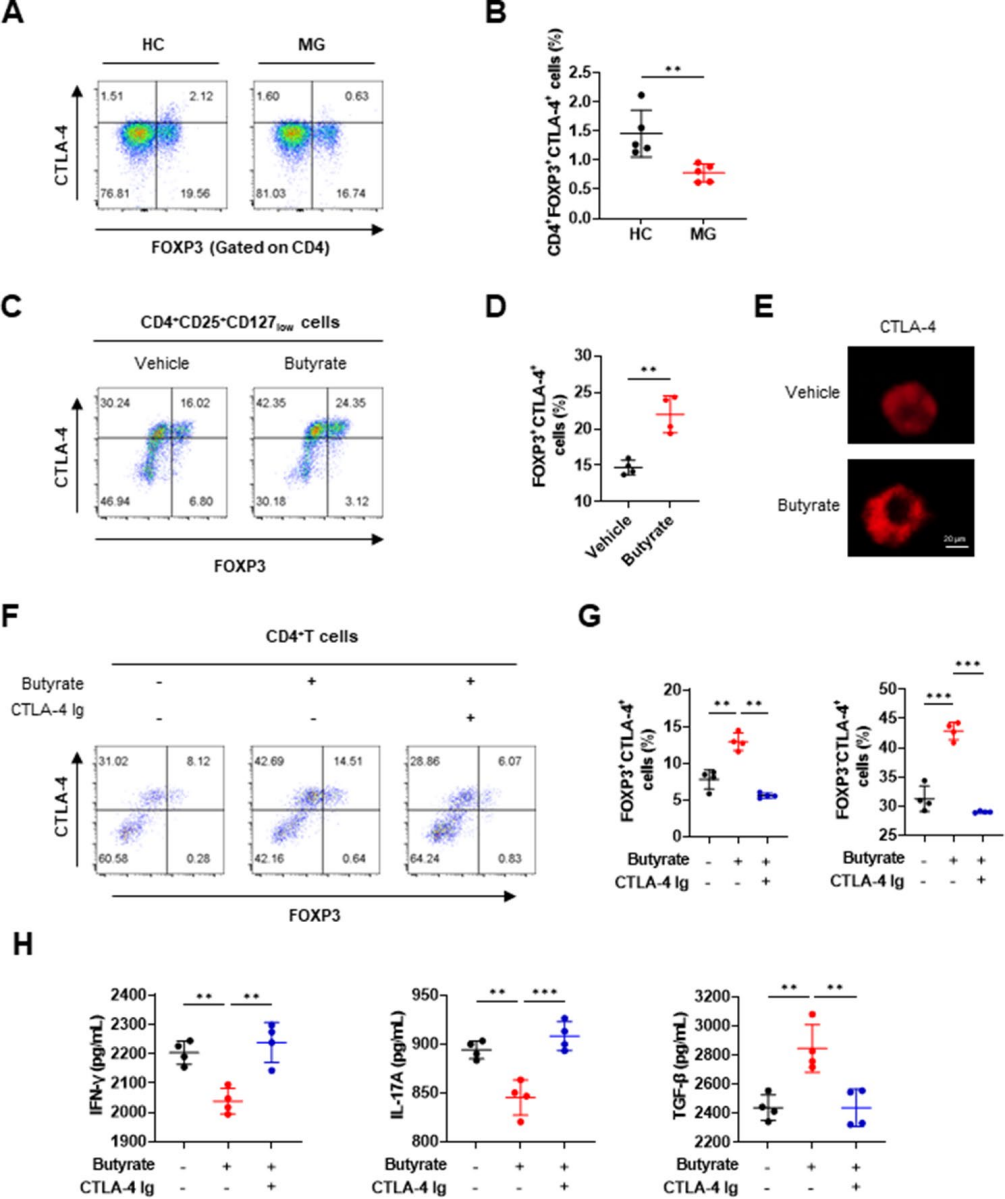
Butyrate enhances surface CTLA-4 expression of Tregs in patients with AChR MG. (**A, B**) The frequencies of surface CTLA-4^+^ in CD4^+^FOXP3^+^ Tregs obtained from the peripheral blood of patients with AChR MG and HCs were determined by flow cytometry (t = 3.525, *p* = 0.0078 with unpaired *t*-test). (**C, D**) Magnetically sorted CD4^+^CD25^+^CD127_low_ Tregs (2 × 10^5^ cells) from patients with AChR MG were cultured for 3 days with or without 200 μM butyrate. The frequencies of CTLA-4^+^FOXP3^+^ cells were determined by flow cytometry (t = 5.357, *p* = 0.0017 with unpaired *t*-test). (**E**) Immunofluorescent images of CTLA-4 on Tregs (scale bar = 20 μm). Magnetically sorted CD4^+^ T cells (2 × 10^5^ cells), obtained from the peripheral blood of patients with AChR MG, were cultured for 3 days under Treg-polarizing conditions with or without CTLA-4-Ig in the presence of 200 μM butyrate. (F, G) The percentages of FOXP3^+^CTLA-4^+^ cells (F = 51.93, *p* = 0.0031 for difference between Butyrate and Butyrate + CTLA-4 Ig with ANOVA) and FOXP3^−^CTLA-4^+^ cells (F = 94.22, *p* < 0.001 for difference between Butyrate and Butyrate + CTLA-4 Ig with ANOVA) were determined by flow cytometry. (**H**) The secretion of IFN-γ (F = 16.85, *p* = 0.0011 for difference between Butyrate and Butyrate + CTLA-4 Ig with ANOVA), IL-17 A (F = 20.96, *p* = 0.0004 for difference between Butyrate and Butyrate + CTLA-4 Ig with ANOVA) and TGF-β (F = 12.99, *p* = 0.0043 for difference between Butyrate and Butyrate + CTLA-4 Ig with ANOVA) in the culture supernatant were quantified using ELISA kits. Each dot represents an individual sample. Data are shown as the mean ± SD, * *p* < 0.05, ** *p* < 0.01, *** *p* < 0.001, ns = not significant

Surface CTLA-4-mediated suppressive function is key for the immunosuppressive function of Treg cells [35], and CTLA-4 is a risk factor in MG [36]. Thus, we evaluated the effects of butyrate on surface CTLA-4 expression in Tregs of patients with MG. In agreement with previous studies, our flow cytometry results showed that surface CTLA-4 expression was significantly lower in CD4^+^ FOXP3^+^ Tregs of patients with AChR MG than in those of HCs (Fig. 4A and B). However, we observed that butyrate supplementation significantly increased the surface CTLA-4 expression of CD4^+^CD25^+^CD127_low_ Tregs in patients with AChR MG (Fig. 4C and D). Immunofluorescence staining also showed that elevated surface CTLA-4 was recruited from intracellular under butyrate treatment (Fig. 4E). Indeed, the treatment of CD4^+^ T cells with butyrate also increased the percentage of FOXP3^+^CTLA-4^+^ cells (Fig. 4F and G). Furthermore, we observed increased levels of TGF-β and reduced levels of IFN-γ and IL-17 A in the cell supernatants after butyrate treatment (Fig. 4H). However, treatment of CD4^+^ T cells with CTLA-4-Ig may block the effects of butyrate on surface CTLA-4 expression and secreted cytokine levels (Fig. 4F–H).

### Butyrate attenuates MG and enhances surface CTLA-4 expression of Tregs in vivo

**Fig. 5:**
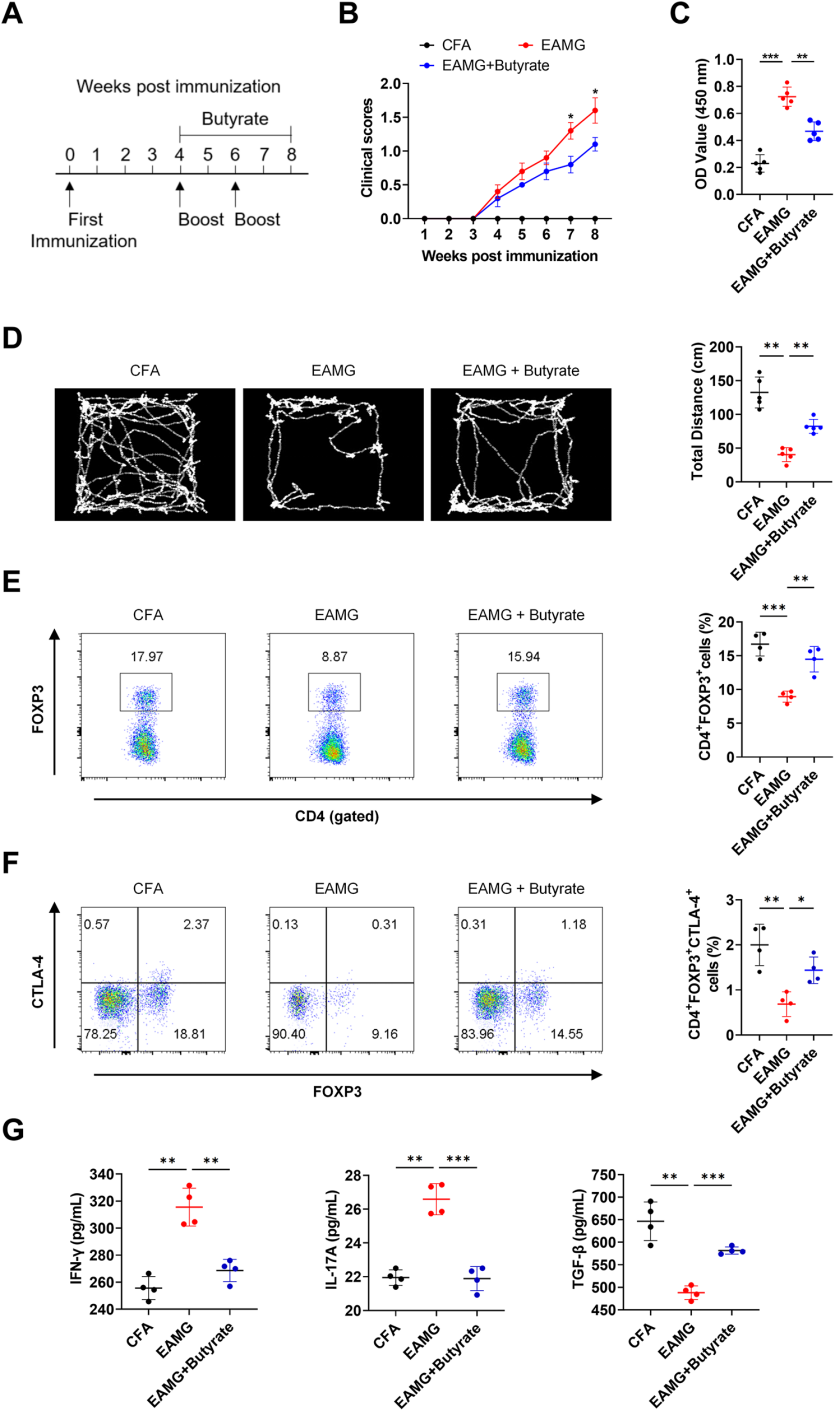
Effects of butyrate on experimental autoimmune MG (EAMG) mice. (**A**) Methods for MG induction in the EAMG mouse model and butyrate administration (*n* = 5). (**B**) Clinical scores of EAMG mice that were or were not treated with butyrate (F = 44.67, *p* = 0.0339 for difference between EAMG and EAMG + Butyrate with ANOVA). (**C**) Levels of serum anti-AChR-Ab antibodies were determined by ELISA from the complete Freund’s adjuvant (CFA) group, EAMG group, and butyrate treatment group (F = 65.63, *p* = 0.002 for difference between EAMG and EAMG + Butyrate with ANOVA). (**D**) Motion tracks for control mice, EAMG mice fed with or without butyrate (F = 55.43, *p* = 0.0023 for difference between EAMG and EAMG + Butyrate with ANOVA). (**E, F**) Percentages of CD4^+^FOXP3^+^ Tregs (F = 26.19, *p* = 0.0019 for difference between EAMG and EAMG + Butyrate with ANOVA) and surface CTLA-4 expression on Tregs (F = 14.05, *p* = 0.0349 for difference between EAMG and EAMG + Butyrate with ANOVA) in the spleen. (**G**) Serum levels of IFN-γ (F = 35.36, *p* = 0.0059 for difference between EAMG and EAMG + Butyrate with ANOVA), IL-17 A (F = 55.81, *p* = 0.0005 for difference between EAMG and EAMG + Butyrate with ANOVA) and TGF-β (F = 35.95, *p* = 0.0003 for difference between EAMG and EAMG + Butyrate with ANOVA) in mice were detected by ELISA. Each dot represents an individual sample. Data are shown as the mean ± SD, * *p* < 0.05, ** *p* < 0.01, *** *p* < 0.001, ns = not significant

Next, we investigated the effects of butyrate on experimental autoimmune MG (EAMG) mice. EAMG mice were immunized with *R97-116* protein and boosted on the 4th and 6th week. After secondary immunization, mice received water or butyrate orally (Fig. 5A). As expected, mice in the butyrate treatment group exhibited lower clinical scores for MG (Fig. 5B), lower serum anti-AChR antibody levels (Fig. 5C), and longer motion tracks than those in the EAMG group (Fig. 5D). In addition, treatment with butyrate increased the percentage of CD4^+^FOXP3^+^ Tregs in the spleen of EAMG mice (Fig. 5E). Surface CTLA-4 expression in Tregs of the spleen also increased under butyrate treatment (Fig. 5F). Moreover, EAMG mice treated with butyrate showed higher levels of TGF-β and lower levels of IFN-γ and IL-17 A in their sera (Fig. 5G).

### Butyrate activates mammalian target of rapamycin (mTOR)-mediated autophagy in Tregs

**Fig. 6:**
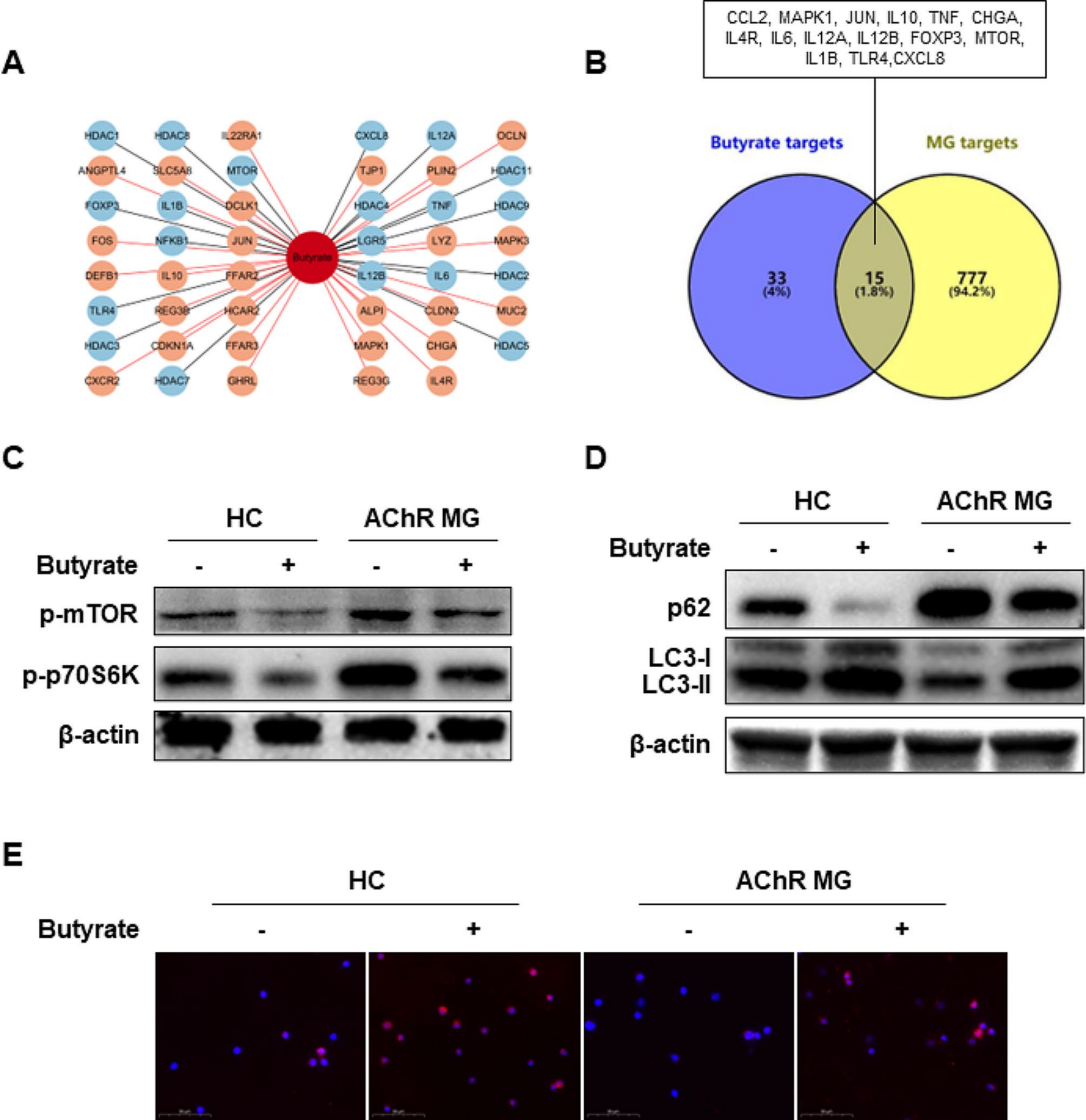
Butyrate activates autophagy by inhibiting mTOR signaling. (**A**) The potential targets of butyrate were identified using the gutMGene database, with positive (red line) and negative (black line) regulation by butyrate. (**B**) A Venn diagram depicting the intersection of MG disease and butyrate targets. (**C, D**) Magnetically sorted CD4^+^CD25^+^CD127_low_ Tregs from HCs and patients with AChR MG were cultured for 5 days with or without 200 μM butyrate. Cells were harvested and the expression of p-mTOR, p-p70S6K, p62, and LC3 was determined by Western blot. (**E**) Immunofluorescent images of LC3 (red) on Tregs (scale bar = 20 μM)

To investigate the underlying mechanism by which butyrate restored the impaired AChR MG Tregs, we used the gutMGene database to identify 48 potential targets of butyrate (Fig. 6A) and the GeneCards database to identify 792 targets of MG. The intersection of these two target sets contained CCL2, MAPK1, JUN, IL10, TNF, CHGA, IL4R, IL6, IL12A, IL12B, FOXP3, MTOR, IL1B, TLR4, and CXCL8 (Fig. 6B). We selected mTOR as a candidate target because it is a major mediator of autophagy [37], which is active in Tregs, and autophagy defects may lead to Treg dysfunction [38]. Indeed, our western blot analysis showed that butyrate treatment reduced the phosphorylation of mTOR and p70S6K in Tregs of HCs and patients with AChR MG (Fig. 6C). This indicated that butyrate could inhibit mTOR signaling in AChR MG Tregs.

To determine whether butyrate restored the impaired Tregs of AChR MG patients by activating autophagy, we measured the expression of p62/SQSTM1 and LC3, markers of autophagy. Western blot analysis revealed that butyrate treatment increased the expression of LC3 proteins and reduced that of p62/SQSTM1 in sorted Tregs from HCs and patients with AChR MG (Fig. 6D). Elevated LC3 expression was also detected using immunofluorescence staining (Fig. 6E). In addition, treatment of naive CD4^+^ T cells under Treg-polarizing conditions with butyrate also reduced the expression of mTOR and enhanced the expression of LC3 (Fig S3A, S3B).

### Butyrate influences Tregs via an autophagy-dependent pathway

**Fig. 7:**
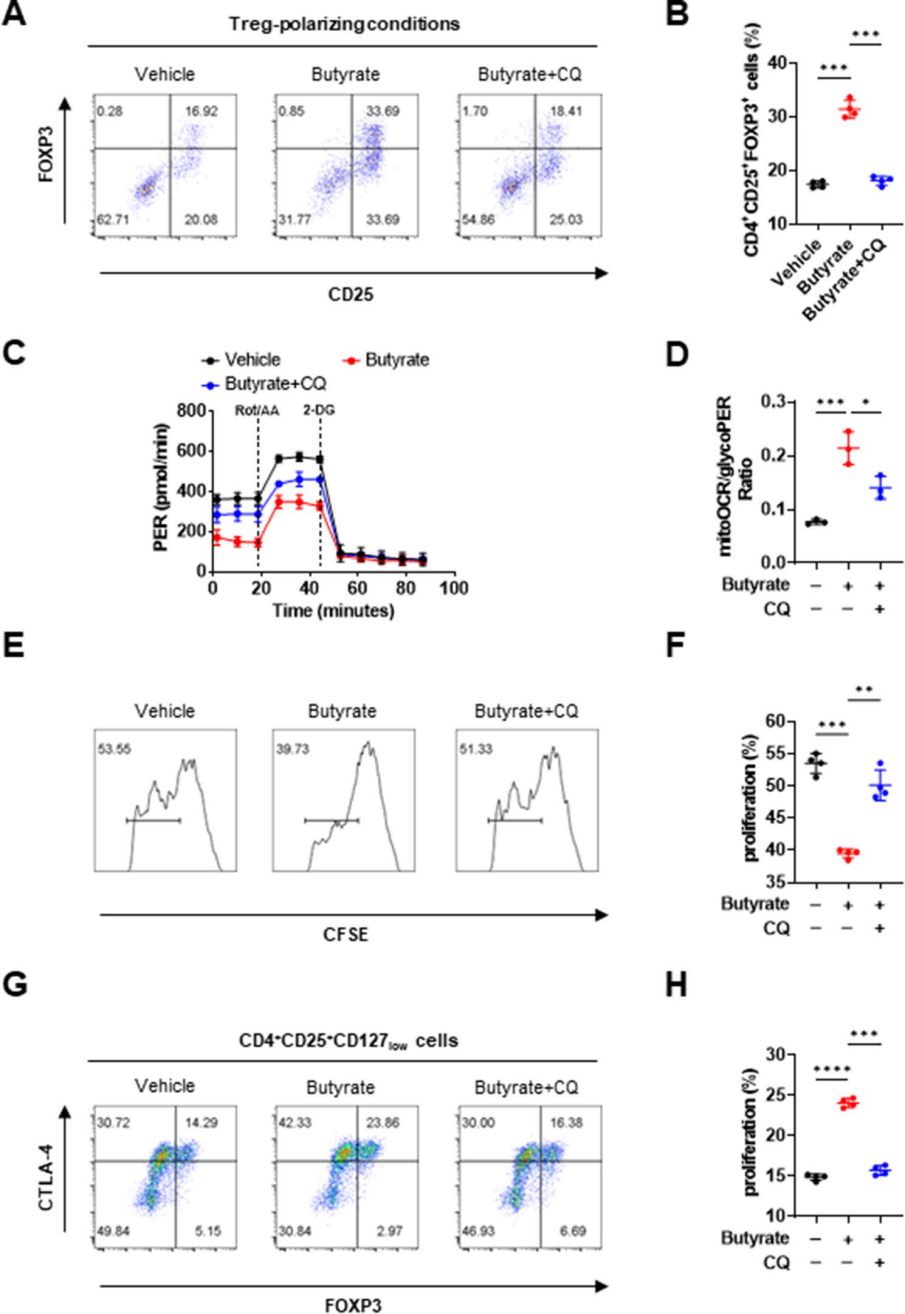
Effects of butyrate on Treg differentiation and function were dependent on autophagy. (**A, B**) Magnetically sorted naive CD4^+^ T cells (2 × 10^5^ cells) from the peripheral blood of patients with AChR MG were cultured for 3 days under Treg-polarizing conditions with or without 200 μM butyrate or 20 μM chloroquine (CQ). The frequencies of CD4^+^CD25^+^FOXP3^+^ Tregs were determined by flow cytometry (F = 203.2, *p* = 0.0003 for difference between Butyrate and Butyrate + CQ with ANOVA). (**C**) The resultant measurement of glycoPER and (**D**) the basal ratio of mitoOCR to glycoPER in these cells (F = 30.57, *p* = 0.0134 for difference between Butyrate and Butyrate + CQ with ANOVA). (**E, F**) Magnetically sorted CD4^+^CD25^−^ Tresps (2 × 10^5^ cells) from patients with AChR MG were labeled with CFSE and cultured for 5 days with or without 200 μM butyrate or 20 μM CQ in an equal number of CD4^+^CD25^+^CD127_low_ Tregs. The suppressive function of Tregs was measured by calculating the proliferation of CFSE^+^ cells (F = 75.07, *p* = 0.0026 for difference between Butyrate and Butyrate + CQ with ANOVA). (**G, H**) Magnetically sorted CD4^+^CD25^+^CD127_low_ Tregs (2 × 10^5^ cells) from patients with AChR MG were cultured for 5 days with or without 200 μM butyrate or 20 μM CQ. The frequencies of CTLA-4^+^ Tregs were detected by flow cytometry (F = 490.8, *p* = 0.0007 for difference between Butyrate and Butyrate + CQ with ANOVA). Each dot represents an individual sample. Data are shown as the mean ± SD, * *p* < 0.05, ** *p* < 0.01, *** *p* < 0.001, ns = not significant

In addition to acting as a mediator of autophagy, mTOR also regulates Treg differentiation through glycolysis [39], and autophagy may be involved in the regulation of glycolysis and CTLA-4 metabolism [39, 40]. Thus, we postulated that butyrate promotes Treg differentiation and suppressive function through the activation of autophagy. Chloroquine (CQ) blocks autophagy by impairing lysosomal function [41], and we observed that CQ significantly blocked the promotion of Treg differentiation by butyrate supplementation (Fig. 7A and B). Inhibition of autophagy also blocked the effects of butyrate on increasing the ratio of mitoOCR/glycoPER in Tregs of patients with MG (Fig. 7C and D). Furthermore, enhancement of Treg suppressive function and surface CTLA-4 expression by butyrate supplementation was significantly reduced by CQ treatment (Fig. 7E–H).

## Discussion

In recent years, gut microbiota has been reported to be involved in the pathology of many autoimmune diseases. MG, an autoimmune disorder of the neuromuscular junction [4], is characterized by impaired Tregs, and it is unclear whether dysregulation of Tregs in patients with MG is associated with their gut microbiota. We showed that gut microbiota-derived butyric acid promotes Treg differentiation and suppressive function in patients with AChR MG. Butyrate supplementation inhibits mTOR signaling, thereby activating the autophagy of Tregs in patients with AChR MG. Furthermore, activated autophagy increases Treg surface CTLA-4 expression and inhibits glycolysis, leading to enhanced Treg suppressive function and differentiation. These results provide a potential mechanism for the link between disturbed gut microbiota and dysregulated Tregs in AChR MG.

The relative abundance of gut microbiota is significantly altered in patients with MG compared with that of microbiota in healthy individual. Zheng et al. observed a decreased OTUs of *Lachnospiraceae*, *Peptostreptococcaceae*, *Ruminococcaceae*, *Erysipelotrichaceae*, and *Clostridiaceae* in MG subjects [23]; Qiu et al. observed decreased relative abundances of *Clostridium* and *Eubacterium* in patients with MG [28], while Morris et al. observed reduced relative proportion of *Verrucomicrobia* and *Actinobacteria* [42]. Furthermore, Liu et al. identified *P. copri*, *Megamonas funiformis*, *Fusobacterium mortiferum*, *Megamonas hypermegale*, and *Prevotella stercorea* as candidate markers for AChR MG patients from HCs [43]. Unlike these studies, we observed a decreased relative abundance of *Clostridia* and *Roseburia* in patients with AChR MG. We hypothesized that differences in illness duration, diet, and living environment all may lead to different characteristic of gut microbiota in patients with AChR MG in different studies.

Gut microbiota constituents, such as *Lachnospiraceae*, and *Erysipelotrichaceae*, can contribute to the production of SCFAs, particularly butyrate [44]. In this study, the relative abundances of *Clostridium* cluster XlVa and *Roseburia* were significantly lower in patients with AChR MG than in HCs. About 95% of SCFAs were absorbed through the intestine before reaching and affecting distant tissues through bloodstream circulation. Compared with the SCFAs in feces, serum butyrate could directly affect Treg differentiation and suppressive function sorted from the peripheral blood of HCs and AChR MG. Thus, we preferred quantified serum level of SCFAs rather than SCFAs levels feces in our study. A quantification of SCFAs in serum indicated a corresponding decrease in butyrate concentrations in patients with AChR MG, which was consistent with its lower concentration in fecal samples [28].

Emerging studies show that butyrate plays an important role in the regulation of Tregs in various diseases; for example, it is involved in promoting the polarization of Tregs in arthritis [29], maintaining the Th17/Treg balance by inhibiting histone deacetylase 1 in inflammatory bowel disease [45], and stimulating bone formation via Treg-mediated regulation of WNT10B expression [24]. We demonstrated that butyrate supplementation effectively increased the differentiation and suppressive function of Tregs in patients with MG. Accordingly, we hypothesized that butyrate may be a key link between the gut microbiota and Treg dysfunction in patients with MG. Besides, we also investigated the effect of butyrate on Tregs in an EAMG mice model. Sun et al. demonstrated that butyrate treatment significantly increased Treg cell numbers, while reduced the presence of Th17, Tfh, and B cells in EAMG mice [46]. We also observed a promotion effect of butyrate on the population of Treg cells in vivo. More importantly, we also found the enhancement of butyrate on surface CTLA-4 expression on Treg cells.

Furthermore, we found that butyrate effectively inhibited the expression of mTOR, which is a key mediator of autophagy [37]. Inhibition of mTOR complex 1 can activate autophagy in Tregs [47], and autophagy is essential for Treg differentiation and function [38, 48]. Thus, we propose that butyrate may reactivate autophagy in Tregs, thereby enhancing Treg differentiation and suppressive functions in patients with MG. Furthermore, surface CTLA-4 expression is reduced in Tregs of patients with MG [11]. Blocking CTLA-4 leads to impaired Treg suppressive function [49]. Moreover, CTLA-4 has been detected in lysosomes [50, 51], indicating that the metabolism of CTLA-4 may be associated with autophagy. We found that butyrate supplementation significantly enhanced autophagy in Tregs of patients with MG, accompanied by increased surface CTLA-4 expression.

mTOR plays a key role in Treg differentiation by regulating glycolysis [39]. Activated autophagy can further suppress glycolysis to maintain Treg stability [39]. We observed that the glycolysis level of Tregs in patients with MG decreased after butyrate treatment. However, whether glycolysis is upregulated or downregulated during Treg differentiation remains controversial. Jun et al. proposed a negative role of glycolysis in Treg differentiation [38], while De Rosa V et al. concluded that the induction of Tregs is dependent on glycolysis [52]. Glycolysis is necessary for early stages of Treg differentiation but is replaced by OXPHOS during later stages to maintain Treg functional stability [53, 54]. We found that butyrate supplementation decreased the level of glycolysis and increased the ratio of mitoOCR/glycoPER in Tregs of patients with MG, indicating a shift from glycolysis to OXPHOS. This suggests that butyrate plays an important role in reprogramming the energy metabolism of Treg differentiation in patients with MG.

Bafilomycin-A1, 3-methyladenine, and CQ are commonly used autophagy inhibitors: bafilomycin-A1 blocks autophagosome-lysosome fusion [55] while 3-methyladenine reduces autophagosome formation [56]. CQ, on the other hand, elevates lysosomal pH, thereby impairing lysosomal enzyme activity [57]. Lysosomes participate in mTORC1-mediated glycolysis and autophagy [64, 65] and may play a role in CTLA-4 degradation [58, 59]. Thus, we selected CQ to investigate whether butyrate promoted AChR MG Treg functions by activating autophagy. We established that CQ treatment blocked the effect of butyrate on Treg differentiation and suppressive function, emphasizing the importance of butyrate-activated autophagy in the dysregulation of AChR MG Tregs.

There remained some limitations in our study. Some researchers did administer butyrate to mice by supplementing into the drinking water [26]. However, it was difficult to measure the amount of butyrate that each mouse assumed. Besides, the quantitative myasthenia gravis (QMG) scores can be applied to assess the disease severity of patients with MG. Since the QMG scores were not obtained in small number of included patients, and we were unable to further analyze the association between QMG scores and relative abundances of gut microbiota in patients with AChR MG, which need our further study in the future.

In summary, our study revealed a link between disturbed gut microbiota and dysregulated Tregs in patients with MG. Butyric acid produced by gut microbiota can effectively promote Treg differentiation and suppressive function by activating mTOR-mediated autophagy. Accordingly, butyrate shows therapeutic potential in the treatment of MG; however, further evaluations are needed in this regard.

### Electronic supplementary material

Below is the link to the electronic supplementary material.

Supplementary Material 16: **Supplementary Fig. 1.** (A) Heatmap of the relative abundance of gut microbiota for candidate markers of AChR MG patients. (B) Receiver operating characteristic (ROC) in the disease classifier. (C) The validation cohort of microbial markers identifies in HCs and patients with AChR (HCs, *n* = 12; AChR MG, *n* = 15)

Supplementary Material 1: **Supplementary Fig. 2.** (A) Naive CD4^+^ T cells obtained from two patients with AChR MG were cultured under Treg-polarizing conditions for 3 days in the presence of 200 μM butyrate; cells were harvested and FOXP3 expression was determined by Western blot. (B) Pearson correlation analysis of serum butyric acid and % Treg in HCs and patients with AChR MG

Supplementary Material 2: **Supplementary Fig. 3.** Magnetically sorted naive CD4^+^ T cells from HCs and AChR MG were cultured for 3 days with or without 200 μM butyrate. (A) The expression of p-mTOR and LC3 were detected by Western blot. (B) LC3 expression was determined by immunofluorescence assay

Supplementary Material 3

Supplementary Material 4

Supplementary Material 5

Supplementary Material 6

Supplementary Material 7

Supplementary Material 8

Supplementary Material 9

Supplementary Material 10

Supplementary Material 11

Supplementary Material 12

Supplementary Material 13

Supplementary Material 14

Supplementary Material 15

## Data Availability

No datasets were generated or analysed during the current study.

## Abbreviations

AChR MG: Anti-acetylcholine receptor antibody-positive myasthenia gravis
AChRs: Acetylcholine receptors
CFSE: Carboxyfluorescein succinimidyl ester
CQ: Chloroquine
CTLA-4: Cytotoxic T-lymphocyte-associated protein 4
EAMG: Experimental autoimmune MG
FOXP3: Forkhead box P3
glycoPER: Glycolytic proton efflux rate
HCs: Healthy controls
MG: Myasthenia gravis
mitoOCR: Mitochondrial oxygen consumption rates
mTOR: Mammalian target of rapamycin
OXPHOS: Oxidative phosphorylation
SCFAs: Short-chain fatty acids
Th: T helper
Treg: Regulatory T
