# Supplementary figures and images for: Gut microbiota-derived butyrate restores impaired regulatory T cells in patients with AChR myasthenia gravis via mTOR-mediated autophagy
Source: Cell Commun Signal. 2024 Apr 3;22:215. doi: 10.1186/s12964-024-01588-9 (PMC10988943; doi:10.1186/s12964-024-01588-9)

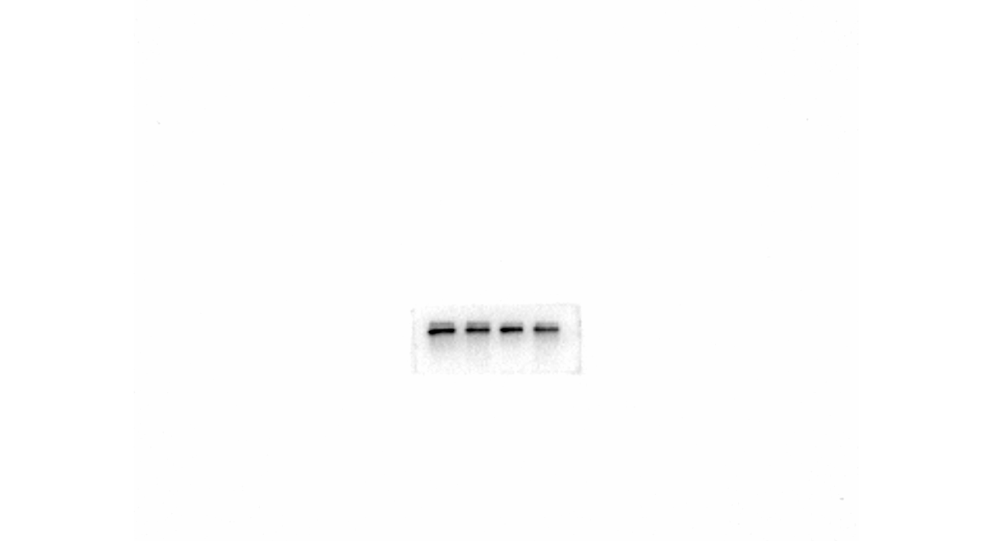

Supplement: Supplementary file 1 — Supplementary Material 16: Supplementary Fig. 1. (A) Heatmap of the relative abundance of gut microbiota for candidate markers of AChR MG patients. (B) Receiver operating characteristic (ROC) in the disease classifier. (C) The validation cohort of microbial markers identifies in HCs and patients with AChR (HCs, n = 12; AChR MG, n = 15) [file 12964_2024_1588_MOESM16_ESM.png]

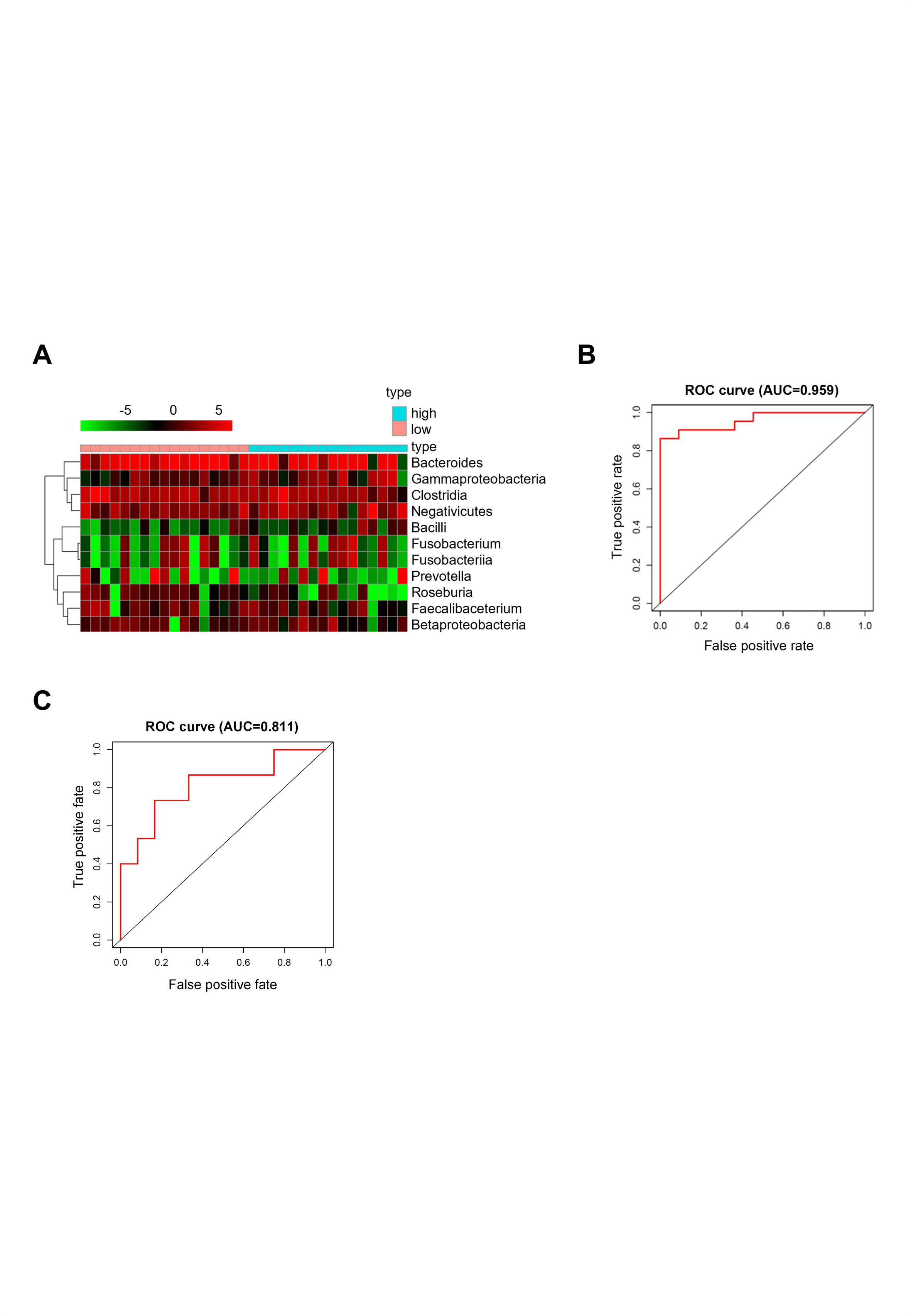

Supplement: Supplementary file 2 — Supplementary Material 1: Supplementary Fig. 2. (A) Naive CD4+ T cells obtained from two patients with AChR MG were cultured under Treg-polarizing conditions for 3 days in the presence of 200 μM butyrate; cells were harvested and FOXP3 expression was determined by Western blot. (B) Pearson correlation analysis of serum butyric acid and % Treg in HCs and patients with AChR MG [file 12964_2024_1588_MOESM1_ESM.png]

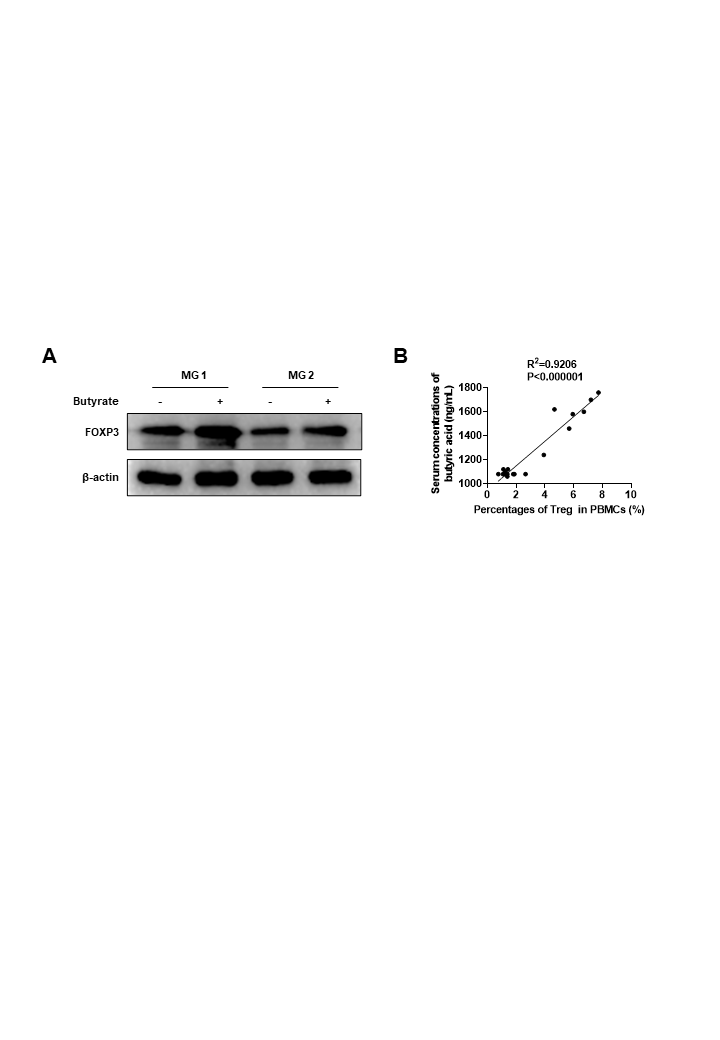

Supplement: Supplementary file 3 — Supplementary Material 2: Supplementary Fig. 3. Magnetically sorted naive CD4+ T cells from HCs and AChR MG were cultured for 3 days with or without 200 μM butyrate. (A) The expression of p-mTOR and LC3 were detected by Western blot. (B) LC3 expression was determined by immunofluorescence assay [file 12964_2024_1588_MOESM2_ESM.png]

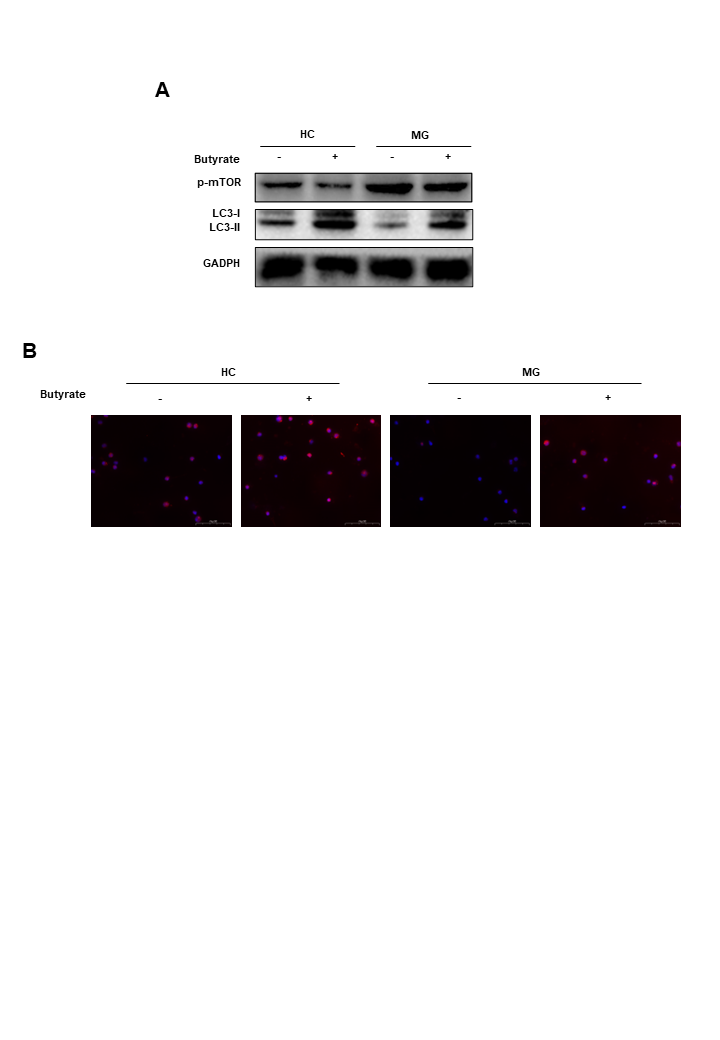

Supplement: Supplementary file 4 — Supplementary Material 3 [file 12964_2024_1588_MOESM3_ESM.png]

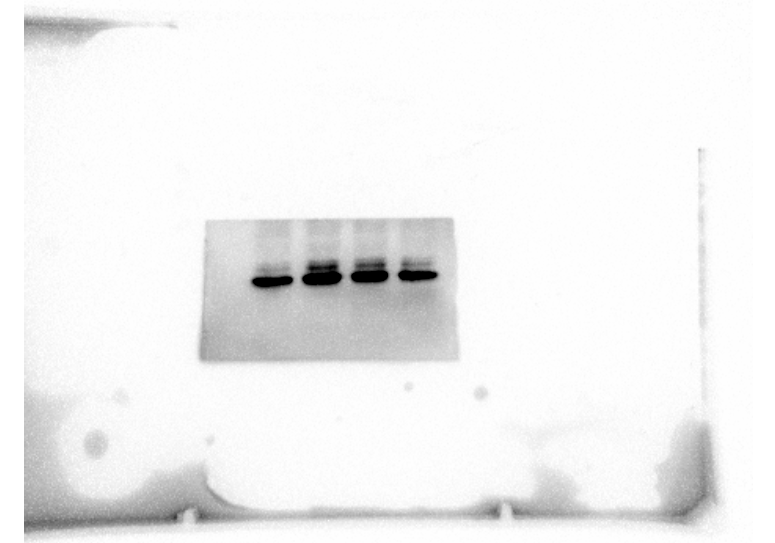

Supplement: Supplementary file 5 — Supplementary Material 4 [file 12964_2024_1588_MOESM4_ESM.png]

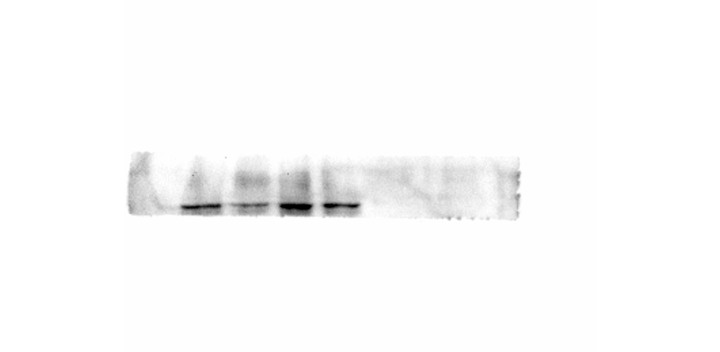

Supplement: Supplementary file 6 — Supplementary Material 5 [file 12964_2024_1588_MOESM5_ESM.png]

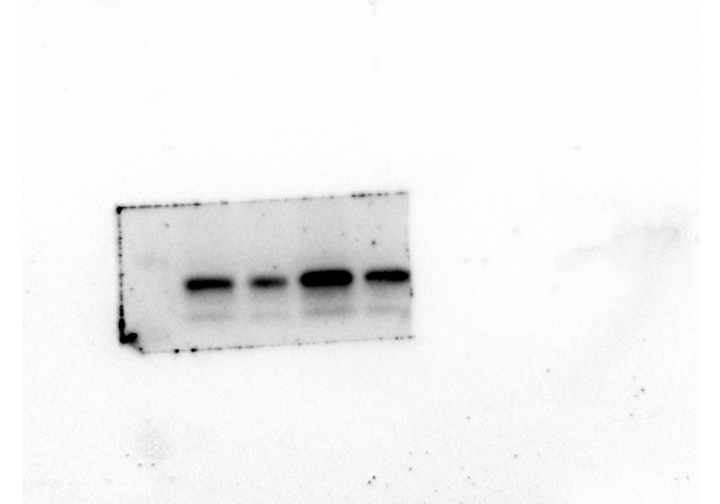

Supplement: Supplementary file 7 — Supplementary Material 6 [file 12964_2024_1588_MOESM6_ESM.png]

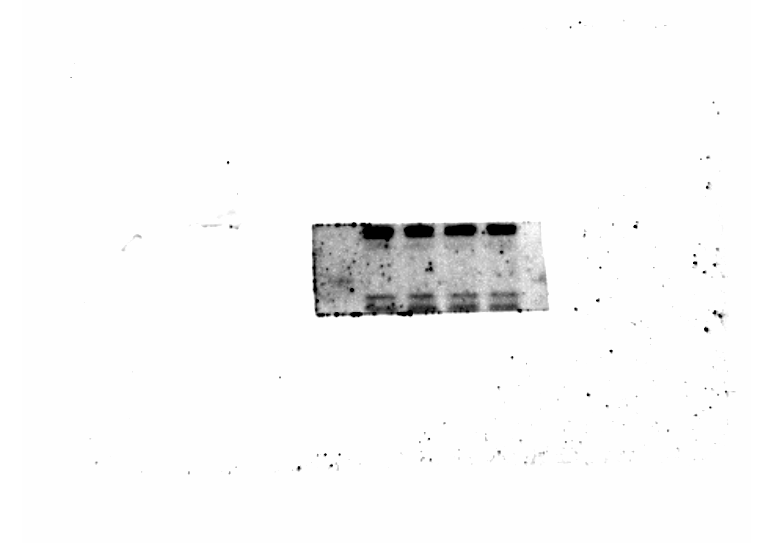

Supplement: Supplementary file 8 — Supplementary Material 7 [file 12964_2024_1588_MOESM7_ESM.png]

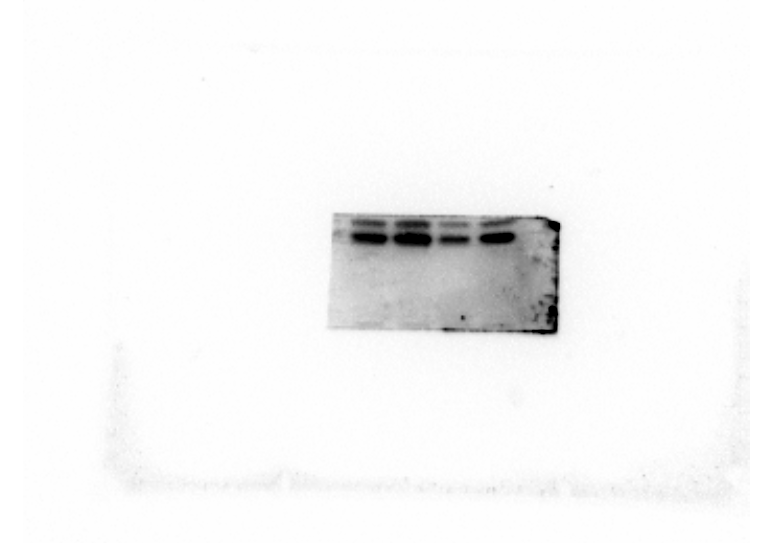

Supplement: Supplementary file 9 — Supplementary Material 8 [file 12964_2024_1588_MOESM8_ESM.png]

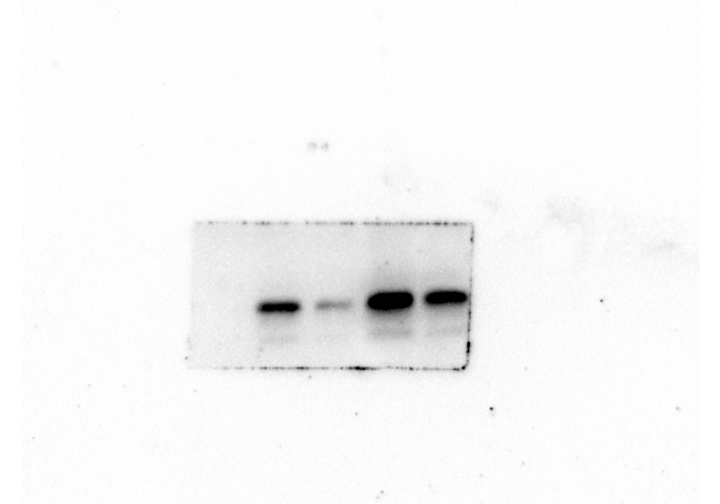

Supplement: Supplementary file 10 — Supplementary Material 9 [file 12964_2024_1588_MOESM9_ESM.png]

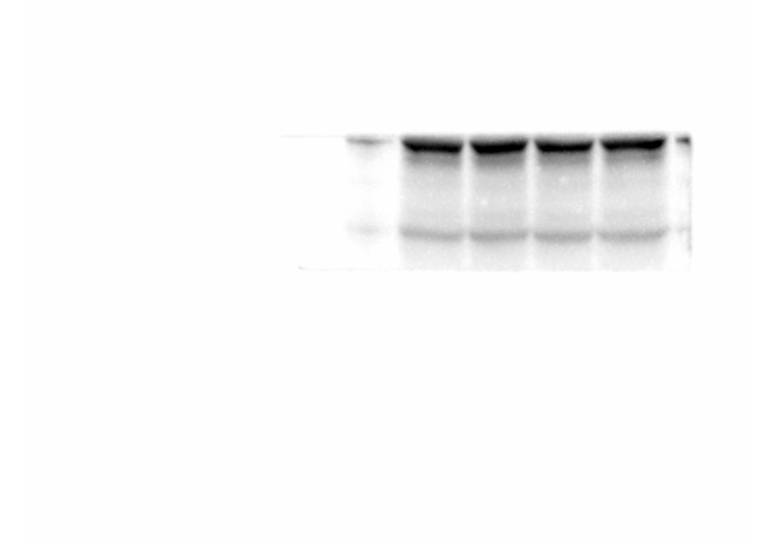

Supplement: Supplementary file 11 — Supplementary Material 10 [file 12964_2024_1588_MOESM10_ESM.png]

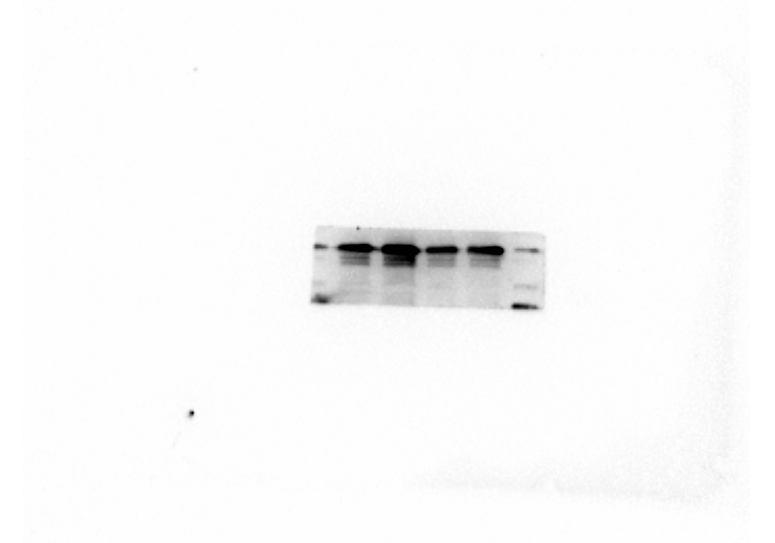

Supplement: Supplementary file 12 — Supplementary Material 11 [file 12964_2024_1588_MOESM11_ESM.png]

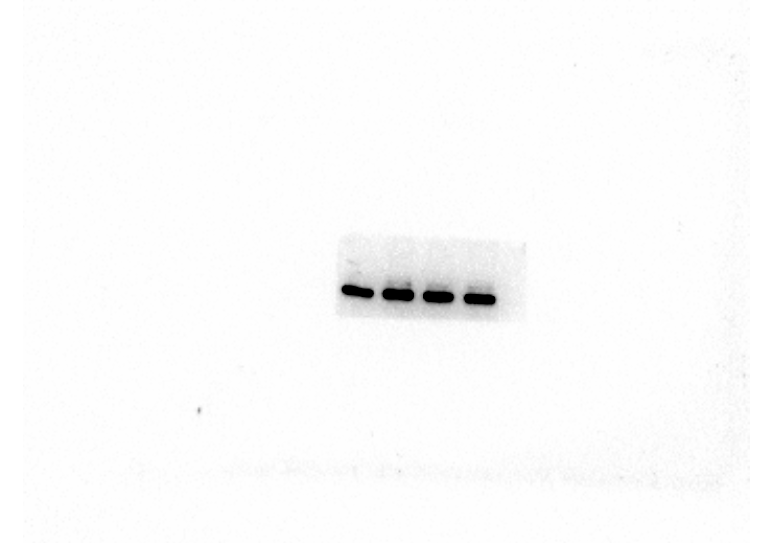

Supplement: Supplementary file 13 — Supplementary Material 12 [file 12964_2024_1588_MOESM12_ESM.png]

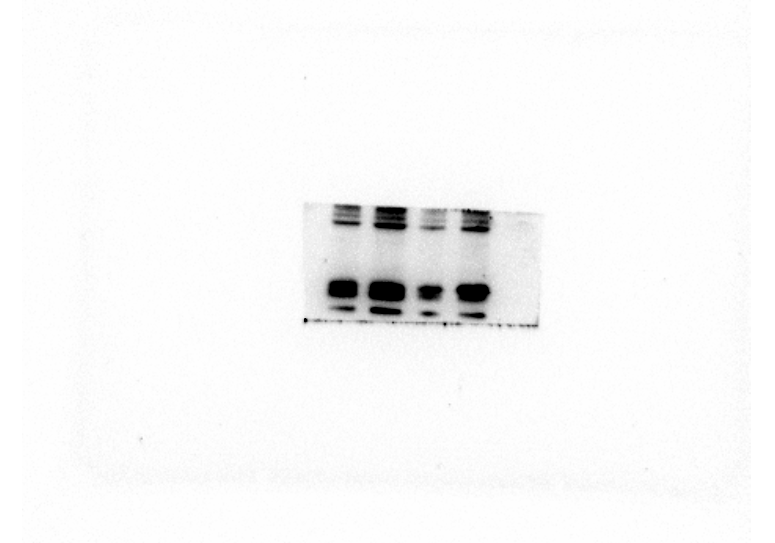

Supplement: Supplementary file 14 — Supplementary Material 13 [file 12964_2024_1588_MOESM13_ESM.png]

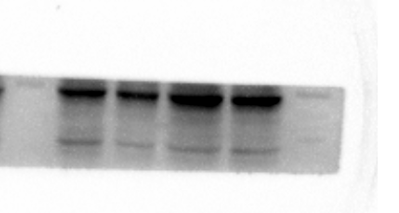

Supplement: Supplementary file 15 — Supplementary Material 14 [file 12964_2024_1588_MOESM14_ESM.png]

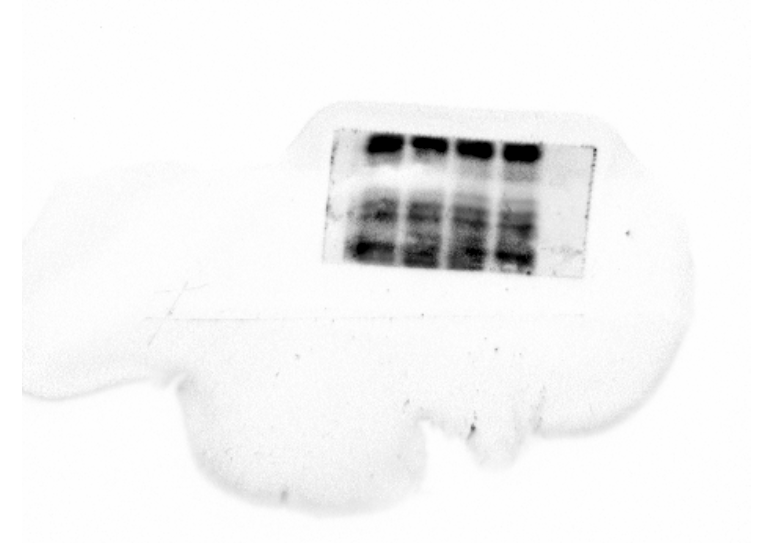

Supplement: Supplementary file 16 — Supplementary Material 15 [file 12964_2024_1588_MOESM15_ESM.png]
